# Salivary Acetylcholinesterase Activity Is Increased in Parkinson's Disease: A Potential Marker of Parasympathetic Dysfunction

**DOI:** 10.1155/2015/156479

**Published:** 2015-02-12

**Authors:** Tatyana Fedorova, Cindy Soendersoe Knudsen, Kim Mouridsen, Ebba Nexo, Per Borghammer

**Affiliations:** ^1^Department of Nuclear Medicine and PET Center, Aarhus University Hospital, 8000 Aarhus, Denmark; ^2^Department of Clinical Biochemistry, Aarhus University Hospital, 8000 Aarhus, Denmark; ^3^Center for Functionally Integrative Neuroscience, Aarhus University Hospital, 8000 Aarhus, Denmark

## Abstract

*Introduction*. Decreased salivary flow and xerostomia are frequent findings in Parkinson's disease (PD), possibly caused by alterations in the parasympathetic tonus. Here we explore salivary acetylcholinesterase (AChE) activity as a potential biomarker in PD. *Methods*. We measured salivary flow, AChE activity, and total protein concentration in 30 PD patients and 49 healthy controls. We also performed exploratory correlation analyses with disease duration, motor symptom severity, autonomic complaints, and other nonmotor symptoms. *Results*. PD patients displayed significantly decreased salivary flow rate, significantly increased salivary AChE activity, and total protein concentration. Importantly, the AChE activity/total protein ratio was significantly increased in PD patients, suggesting that increased AChE activity cannot be explained solely by upconcentration of saliva. The Unified PD Rating Scale (UPDRS) score displayed significant correlation with total salivary protein (*P* = 0.002) and near-significant correlation with salivary flow (*P* = 0.07). Color vision test scores were also significantly correlated with AChE activity (*P* = 0.04) and total protein levels (*P* = 0.002). *Conclusion*. Salivary AChE activity is increased in PD patients compared to healthy controls. Future studies are needed to elucidate whether this parameter reflects the extent of neuronal damage and parasympathetic denervation in the salivary glands of PD patients.

## 1. Introduction

**Figure 1:**
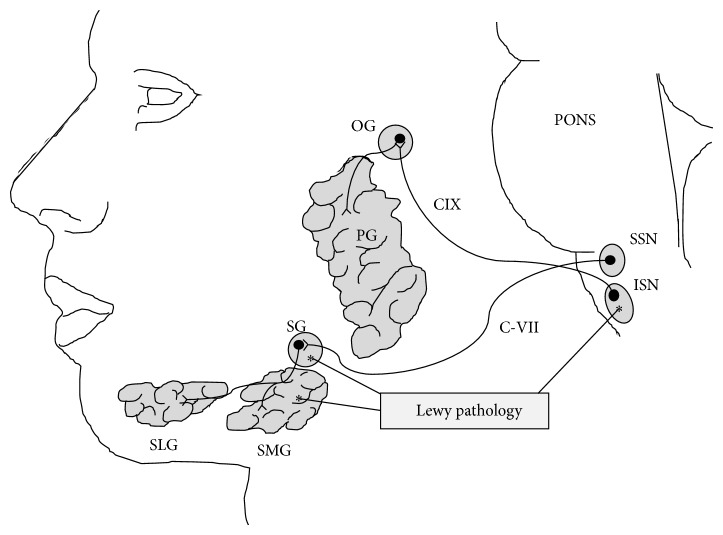
The preganglionic parasympathetic cell bodies responsible for innervation of the submandibular and sublingual glands are located in the superior salivary nucleus and terminate in the submandibular ganglion, from which postganglionic fibres reach the glands. The preganglionic cell bodies which innervated the parotid gland are located in the inferior sal nucleus and terminate in the otic ganglion. Lewy body pathology has been detected in the submandibular gland, submandibular ganglion, and inferior salivary nucleus. (C-VII, facial nerve; CIX, glossopharyngeal nerve; ISN, inferior salivatory nucleus; OG, otic ganglion; PG, parotid gland; SG, submandibular ganglion; SLG, sublingual gland; SMG, submandibular gland; SSN, superior salivatory nucleus).

Parkinson's disease (PD) is a disabling, multisystem disorder characterized by progressive degeneration of monoaminergic and cholinergic neurons in the brain stem and basal forebrain [1]. Recently, phosphorylated *α*-synuclein (*α*Syn), the biological hallmark of alpha-synucleinopathies, has also been demonstrated in the spinal cord, cerebrospinal fluid, cardiac nervous system, myenteric and submucosal plexus of the enteric nervous system, vagal nerve, and parasympathetic ganglia [2–4]. Importantly, the submandibular glands contain *α*Syn pathology in nearly all PD patients [3, 5]. Xerostomia and decreased salivary flow in PD patients have been reported by several authors [6, 7], and this nonmotor symptom is probably related to the burden of *α*Syn pathology in the glands (Figure 1). Parasympathetic denervation of the salivary glands or even colocalization of *α*Syn pathology with cholinergic, parasympathetic terminals has, however, not been convincingly demonstrated. Thus, our understanding of the pathological substrate of xerostomia and decreased salivary flow remains incomplete.

Saliva is a readily available biofluid, which, compared to blood and cerebrospinal fluid (CSF), can be obtained noninvasively. The development of a successful salivary biomarker for PD has the potential to be of major importance for diagnostics, the monitoring of disease progression, and therapeutic interventions. A recent study of PD patients demonstrated that levels of salivary *α*Syn may be decreased, and salivary DJ-1 protein increased in PD [8]. Another study reported increased levels of salivary amylase in PD patients compared to controls [6]. Furthermore, a study of salivary cortisol levels in PD patients showed increased levels of cortisol in patients without impulsive compulsive behavior compared with controls [9]. These encouraging results signify that development of salivary biomarkers of PD may be feasible.

Acetylcholinesterase (AChE) hydrolyzes the neurotransmitter acetylcholine (ACh). Large quantities of AChE are synthesized in the perikaryon of cholinergic neurons and transported to dendrites and axons, where it is secreted into the synaptic cleft [10]. AChE has therefore been considered a potential biomarker of cholinergic function in general, and blood AChE levels were utilized in biomonitoring of pesticide exposure [11, 12]. Altered levels of salivary AChE activity have also been detected in patients exposed to pesticides, and this approach has recently been explored as an alternative to blood measurements in monitoring pesticide exposure [13]. Furthermore salivary AChE activity has been proposed as a potential test for central cholinergic function in patients with Alzheimer's disease [14]. To our knowledge, salivary AChE activity has not been assessed in a population of PD patients, despite the clear signs of parasympathetic denervation in this disorder.

In the present exploratory study, we investigated salivary AChE activity as a potential biomarker in PD. We measured salivary flow, AChE activity, and total protein concentration in PD patients and a healthy control group. We hypothesized that these parameters would be altered in the PD group. Furthermore, exploratory correlation analyses with clinical parameters, including disease duration, motor symptom severity, autonomic complaints, and other nonmotor symptoms, were performed.

## 2. Methods

### 2.1. Subjects

We enrolled 30 PD patients from the movement disorder clinic at the Department of Neurology, Aarhus University Hospital. A total of 49 age-matched controls were recruited by newspaper advertisement. The study was conducted in accordance with the declaration of Helsinki and was approved by the review board of the regional science ethics committee. All subjects provided written consent prior to enrollment in the study. The diagnoses of PD were made by movement disorder specialists according to the U.K. Parkinson's Disease Society Brain Bank criteria [15].

The PD patients received the following medication: levodopa (*n* = 4), levodopa+agonist (*n* = 10), levodopa+MAO-B inhibitor (*n* = 1), levodopa+amantadine (*n* = 1), agonist (*n* = 6), agonist+MAO-B inhibitor (*n* = 2), levodopa+MAO-B inhibitor+agonist (*n* = 2), and no medication (*n* = 4). Exclusion criteria included serious medical conditions (severe disorders of the heart, lungs, liver, and kidneys), neurological disorders, and diseases affecting the autonomic function or the salivary glands such as Sjögren's disease, cancer of the head and neck, or radiation therapy to the head and neck region. Subjects treated with drugs that may affect the function of AChE (donepezil, physostigmin, pyridostigmin, neostigmin, rivastigmin, and galantamin) were excluded.

### 2.2. Salivary Flow and Biochemical Studies

Samples of whole saliva were obtained from PD patients and healthy volunteers using a standardized saliva collection method [14, 16]. All participants had refrained from food, smoking, and beverages other than water for a minimum of four hours. PD patients were off all PD medication for a minimum of 12 hours. The subjects were seated in a chair and asked to rinse their mouth with still mineral water. They were instructed to spit all produced saliva into a preweighted test tube and not to swallow or talk during the collection. Saliva collected during the first five minutes was discarded and only that obtained during the following 10–50 minutes was used for further analyses. Samples were kept on ice immediately after collection and during subsequent initial processing to minimize degradation of salivary proteins. Flow rate was calculated as g/min, which is nearly equivalent to mL/min [16]. Samples were centrifuged at 3000 rpm for 30 minutes to remove particulate matter. The supernatant was divided into aliquots of 500 *μ*L, which were sonicated for 3 × 10 seconds, and then immediately frozen at −80°C for a maximum of 7 months until further analyses.

Total salivary protein (TP) concentration was determined using a colorimetric assay, bicinchoninic acid (BCA) protein assay according to the manufacturer's protocol in a 96-well microplate (Thermo Scientific, IL, USA). The plates were read at 540 nm using a Multiscan Ascent 96/384 plate reader (MTX Lab Systems Inc., Virginia, USA).

The catalytic activity of AChE was determined by the colorimetric method [17], used on salivary samples with slight modifications [13]. For each measurement, 100 *μ*L of saliva and 25 *μ*L of 10 mM dithionitrobenzene (DTNB) (Sigma-Aldrich, Missouri, USA) were added to 750 *μ*L of 0.1 M phosphate buffer (pH = 8). This mixture was incubated for 10 minutes and 40 *μ*L of 75 mM acetylthiocholine iodide (Sigma) was added to initiate the reaction. Samples were incubated for 90 minutes at 37°C and the reaction was terminated by adding 25 *μ*L of 12 mM eserine salicylate (Sigma-Aldrich) to all samples. Each analytical batch had one blank sample and one control (pooled red blood cells). All samples, controls, and blanks were analyzed in duplicate. Changes in color intensity were measured at 405 nm using the Multiscan plate reader. Enzyme activity is expressed as absorbance units (a.u.) and normalized to the sample protein content (AChE activity/TP ratio). Salivary AChE activity measurements in three healthy control samples and total protein measurements in one healthy control sample failed due to insufficient amounts of saliva.

### 2.3. Clinical and Other Investigations

Motor symptoms in the PD patient group were rated using the Unified Parkinson Disease Rating Scale (UPDRS III) motor score [18]. Clinical stage was rated with the Hoehn and Yahr (HY) stage [19]. The Mini-Mental State Examination (MMSE) was used to screen for dementia symptoms. Olfactory function was investigated using the validated 16-item “Sniffin' Sticks” smell-identification battery [20].

We performed color vision testing using the Farnsworth-Munsell 100 Hue Test [21], in which participants are asked to sort 85 randomly mixed colored discs according to four-color spectra since we hypothesized that poor color vision and loss of cholinergic neurons may be related.

Full UPDRS motor scores were not obtained in three patients. Olfactory testing and color vision testing were not performed in one healthy subject due to nausea. Color vision results from one healthy subject and two PD patients were excluded from further analyses due to color blindness.

Symptoms of autonomic dysfunction were assessed using questionnaires. ROME-III was used for assessing GI symptoms. We used the Constipation Module and calculated both the total score and the isolated sum of the constipation items (questions 9–14) [2, 22]. We investigated sleeping habits using the REM sleep behavior disorder screening questionnaire (RBDSQ) [23]. Salivary symptoms were evaluated using the Drooling Rating Scale (DRS) [24]. We did not attain DRS results from two healthy controls and one PD patient.

### 2.4. Data Analysis

Statistical analyses were performed using R-software (The R Foundation for Statistical Computing, Vienna, Austria). Between-group differences were calculated for parametric data using two-tailed Student's *t*-test and for nonparametric parameters using Chi-square test. We examined possible correlations between disease duration, UPDRS, olfaction, color vision, and constipation scores and salivary parameters, that is, flow, AChE activity, total protein concentration, and AChE/TP ratios using *F*-test for linear regression. Adjustments for age and gender were made using multiple linear regression. Numerical values are expressed as mean ± SD. As this was an exploratory study, we did not perform corrections for multiple comparisons.

## 3. Results

Demographic and clinical scores are summarized in Table 1. No significant differences in age, gender, and MMSE-scores were found between groups.

### 3.1. Saliva Studies

**Figure 2:**
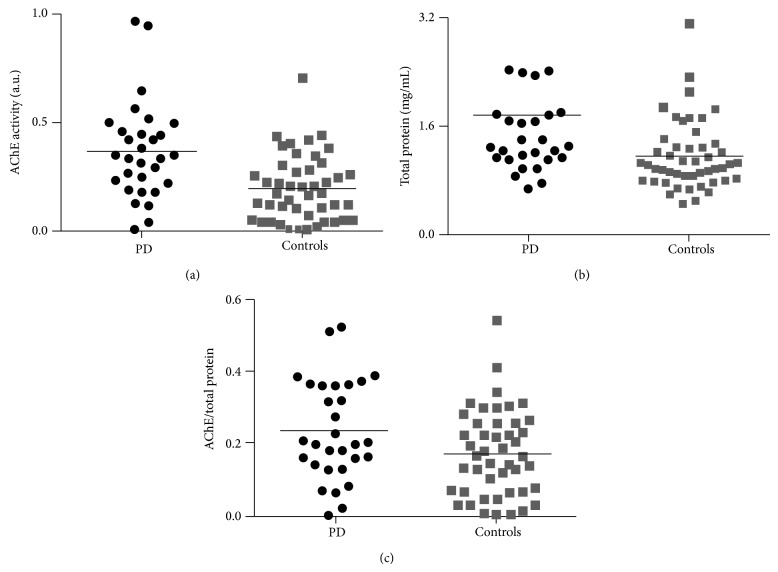
Salivary AChE activity (a), TP concentration (b), and AChE/TP ratio (c) in 30 PD patients and 49 control subjects. (Three TP outliers in the PD group not shown on graph).

Results from the salivary studies are displayed in Figure 2 and Table 2. The PD group displayed significantly decreased salivary flow rate and significantly increased AChE activity and TP concentration compared to controls. Our data suggested that some AChE and TP values could be statistical outliers, we tested these using robust regression and outlier removal (ROUT) [25]. In the TP data, three PD and one control value were significant outliers. The between-group difference in TP remained significant after removal of these outliers (*P* = 0.0047). No significant outliers were detected in the AChE data, and removing the two extreme PD AChE values did not influence the level of significance (*P* = 0.0008).

Importantly, the AChE/TP ratio was significantly increased in PD patients, signifying that the increased AChE activity could not be explained solely by generalized increased protein concentration. After adjusting for age and gender, using a multiple linear regression, these differences between patients and controls remained significant (*P* = 0.04). After exclusion of the four significant TP outliers, the AChE/TP difference also remained significant (*P* = 0.014).

### 3.2. Clinical Investigations and Correlations

**Figure 3:**
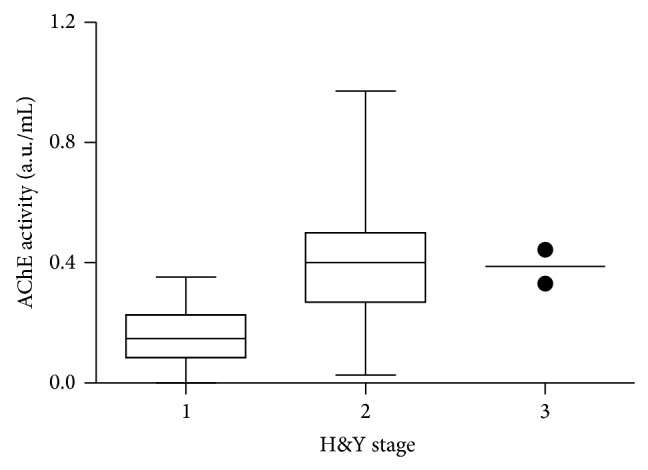
Box-and-whiskers plot of AChE activity in the H&Y subgroups. The plot shows mean, 25th and 75th percentiles, minimum, and maximum values. The H&Y stage 3 group included only two samples that are represented as dots.

Questionnaires, olfactory, and color vision tests are summarized in Table 2. Significant between-group differences were detected in all investigated parameters (*P* < 0.05). The UPDRS scores displayed significant correlation with total salivary protein (adjusted *r* = 0.54; *P* = 0.002) and near-significant correlation with salivary flow (adjusted *r* = 0.3; *P* = 0.07). UPDRS showed no correlations with AChE activity and AChE/TP ratios. In contrast, ANOVA tests of H&Y stage subgroups (Figure 3) demonstrated significantly different AChE salivary activity levels among stages (*P* = 0.035). For the normalized AChE/TP ratios, this difference was only borderline significant (*P* = 0.08).

In the PD group, significant positive correlations between age and AChE activity (adjusted *r* ^2^ = 0.37; *P* = 0.0002) as well as salivary TP (adjusted *r* = 0.61; *P* = 0.02) were seen. In the control group, these correlations were weaker, that is, age versus TP (adjusted *r* = 0.24; *P* = 0.06) and age versus AChE activity (adjusted *r* = 0.2; *P* = 0.11).

Interestingly, correlation was seen between color vision test scores and AChE activity (adjusted *r* = 0.33; *P* = 0.04) and TP levels (adjusted *r* = 0.54; *P* = 0.002), but not with the normalized AChE/TP ratios (*P* = 0.65). No correlation was found in the PD group between color vision test scores and the RBDSQ results (*P* = 0.99).

Disease duration, olfaction scores, constipation scores, and sleeping habits did not exhibit significant correlation with salivary parameters, that is, flow, AChE activity, or AChE/TP ratios.

## 4. Discussion

Our finding of significant hyposialorrhea in the PD group is in accordance with previous studies documenting decreased salivary flow as a clear and early manifestation of autonomic dysfunction in PD [6, 7]. Total salivary protein content has not previously been explored in the PD, but our finding of increased TP concentration is consistent with previous findings of increased amylase levels in saliva from PD patients [6].

To our knowledge, this is the first investigation of salivary AChE activity levels in PD. Significantly increased levels of AChE activity were seen in the PD group. Patients in H&Y stages 2-3 displayed higher levels of AChE activity than stage 1 patients, although AChE activity did not correlate with UPDRS motor scores. Increased salivary TP was also seen in the patient group suggesting that saliva becomes more concentrated, as the volume decreases. When normalizing AChE activity to salivary TP, the AChE/TP ratio was still increased in the patient group. Thus, AChE activity seems to be increased beyond what can be explained by mere concentration of saliva. However, considerable overlap was seen in AChE and AChE/TP results between the two groups. This makes AChE activity measurements unsuitable as a clinical diagnostic biomarker at present, but the technique could have potential for elucidating the pathophysiology of parasympathetic denervation in PD.

It is well known that Lewy pathology is almost universally present in the dorsal motor nucleus of the vagal and glossopharyngeal nerves [26]. Alpha-synuclein pathology was also detected in the peripheral motor and sensory nerves innervating the laryngopharynx in PD patients [27, 28]. Lewy bodies have been reported in the submandibular ganglion [29] and also in the inferior salivatory nucleus (Del Tredici et al., 2002), although the latter may be an infrequent finding (Figure 1). To our knowledge, no studies have investigated the presence of pathological alpha-synuclein aggregations in the superior salivatory nuclei or the parasympathetic. Future studies are needed to investigate correlations between* in vivo *symptoms related to salivation and the presence of neurodegeneration in the relevant brainstem and ganglionic structures.

We hypothesize that increased salivary AChE activity could be caused by progressive *α*Syn deposition and consequent neuronal damage to the glands' parasympathetic nerve terminals. This would be similar to the increased CSF tau protein levels present in patients with Alzheimer's disease (AD), thought to be caused by neuronal cell death and consequent release of tau [30]. A recent study performed transcutaneous needle core biopsies of submandibular glands in PD patients and demonstrated pathological *α*Syn depositions in the majority of patients [5]. Future studies could investigate the correlation between salivary parameters such as AChE activity and immunohistochemical staining of *α*Syn burden in the glands and the colocalization of *α*Syn with cholinergic (parasympathetic) nerve fibers.

Interestingly, patients with Sjögren's syndrome also exhibit increased salivary levels of cholinesterase activity [31]. In Sjögren's syndrome, nerve fibers are degenerated and a general decrease in number of nerve fibers is seen in the labial glands [32].

A significant association between H&Y stage and salivary AChE levels was detected, but no correlation was seen with the UPDRS motor scores. Thus, in general the association between clinical severity of PD and salivary AChE is probably weak. Furthermore, our data set included only six patients in H&Y stage 1. As such, this result needs to be reproduced in future studies of independent and larger patient populations, including both very early-stage and late-stage patients. Such studies could potentially also investigate correlations between individual UPDRS III items and salivary parameters.

Poor color vision correlated inversely with both AChE activity and TP concentration. Previous studies demonstrated that PD patients with RBD exhibit poorer color vision and a greater loss of cholinergic innervation in the brain compared to PD patients without RBD [33]. Those observations suggest that loss of color vision may be associated more with damage to cholinergic neurons. Our observed inverse correlation may suggest that poor color vision could be associated not only with loss of cerebral cholinergic function, but also with peripheral cholinergic damage in parasympathetic nerves. We did not, however, see any significant correlation between color vision and the RBDSQ scores, but it was recently pointed out that the RBDSQ scale is probably not optimal for RBD evaluation in PD and that polysomnography should still be considered the gold standard for this diagnosis [34].

This study has some limitations. The PD patient group included a limited number of patients in the earliest disease stage. Thus, although a correlation between H&Y stage and AChE activity was suggested, this observation was based on only six H&Y stage 1 patients. Any association between disease stage and salivary AChE activity levels needs to be reproduced before firm conclusions can be drawn. We did not perform multiple comparison correction. When using Bonferroni correction the group differences in TP and nonnormalized AChE levels remained significant, whereas the normalized AChE/TP ratio was not. However, there is no universal agreement concerning the use of Bonferroni correction, and many statisticians advocate against it [35]. As this was an exploratory study, we did not perform correction for multiple comparisons but specified the exact *P* value for each test.

The measured activity levels of AChE in saliva are low and thus we anticipated that a more accurate estimate could be obtained by measurement of the protein rather than the activity of AChE. We attempted to do so, using several different commercially available enzyme-linked immunosorbent assays (ELISA), but this approach proved unsuccessful due to poor sensitivity of the assays and unacceptably high variation among different ELISA plates (data not shown).

In summary, the present study demonstrated increased AChE activity in saliva from PD patients compared with healthy controls. Significant positive correlations were seen in the PD group between AChE activity and H&Y stage. Disease duration, olfaction scores, constipation scores, and sleeping habits did not exhibit significant correlations with salivary parameters. Interestingly, we detected a significant correlation between color vision test scores and AChE activity and TP levels. Future studies are needed to address possible associations between the salivary parameters and the extent of neuronal damage in the salivary glands.

## Figures and Tables

**Table 1:** Clinical and demographic characteristics of patients and controls.

	Controls (*n* = 49)	PD patients (*n* = 30)	*P* value
Age	62.7 ± 9.4	63.7 ± 9.1	0.6
Sex (M/F)	27/22	16/14	0.88
MMSE	28.9 ± 1.1	28.6 ± 1.5	0.5
UPDRS III	N/A	27 ± 12.8	N/A
H&Y stage	N/A	1.9 ± 0.5	N/A
Duration since PD diagnosis (years)	N/A	4.8 ± 3.3	N/A

All values are mean ± SD.

**Table 2:** Comparison of clinical and biochemical parameters in patients and controls.

	Controls (*n* = 49)	PD patients (*n* = 30)	*P* value
Sniffin' Sticks	11.1 ± 2.7	6.8 ± 2.6	**<0.001**
FM100 error score	63 ± 41.1	89 ± 56.2	**0.03**
RBDSQ	2.5 ± 2.3	4.3 ± 2.9	**0.005**
ROME III-LGI	4.1 ± 5.9	11.5 ± 11.4	**0.002**
ROME III-constipation	1.5 ± 2.3	5.6 ± 5.3	**<0.001**
DRS	0.6 ± 1.6	2.6 ± 3.1	**<0.001**
Salivary flow (mL/min)	0.36 ± 0.2	0.25 ± 0.2	**0.02**
Total protein (mg/mL)	1.2 ± 0.5	1.8 ± 1.1	**0.002**
AChE activity (a.u.)	0.19 ± 0.15	0.36 ± 0.22	**<0.001**
AChE/total protein ratio (a.u./mg)	0.17 ± 0.12	0.23 ± 0.14	**0.04**

ROME III-LGI: lower GI symptoms questionnaire; ROME III-constipation: questions 9–14 from ROME III-LGI. All values are mean ± SD.
